# Predictive modeling of aneurysmal subarachnoid hemorrhage with acute lung injury complicating delayed cerebral ischaemia

**DOI:** 10.3389/fneur.2025.1535654

**Published:** 2025-03-12

**Authors:** Chang Su, Jianping Ye, Jin Liu

**Affiliations:** ^1^Department of Neurosurgery, Lishui Hospital of Wenzhou Medical University, Lishui, China; ^2^Department of Intensive Care Unit, Lishui Hospital of Wenzhou Medical University, Lishui, China

**Keywords:** aneurysmal subarachnoid hemorrhage, acute lung injury, delayed cerebral ischaemia, columnar line drawing, predictive model

## Abstract

**Objective:**

Delayed cerebral ischemia (DCI) is a frequent consequence of aneurysmal subarachnoid hemorrhage (aSAH), and severe aSAH is typically accompanied with Acute Lung Injury (ALI). This research examined the risk variables for delayed cerebral ischaemia in aneurysmal subarachnoid hemorrhage patients complicated with ALI, and developed a columnar graph prediction model.

**Methods:**

Clinical data from 234 patients with aSAH complicated with ALI, admitted to Lishui People’s Hospital between January 2018 and June 2024, were analyzed. The patients were randomly divided into a training group (164 cases) and a validation group (70 cases). Risk factors for the occurrence of delayed cerebral ischaemia (DCI) were identified and incorporated into a model, the differentiation and reliability of the line graph model were validated via the use of ROC curves and calibration curves.

**Results:**

Multifactorial logistic regression identified three significant independent risk variables for DCI: elevated positive end-expiratory pressure (PEEP), interleukin-6, and D-dimer (*p* < 0.05). The column-line plots demonstrated superior discriminatory performance in both the training set (AUC = 0.882, 95% CI: 0.820–0.940) and the validation set (AUC = 0.874, 95% CI: 0.778–0.996), while the calibration curves indicated strong concordance between the training and validation sets.

**Conclusion:**

High positive end-expiratory pressure, interleukin-6, and d-dimer are independent risk factors for DCI in patients with aSHA combined with ALI, and the resulting columnar line graphs show significant predictive value and help to better identify patients at high risk of DCI.

## Introduction

Accounting for morbidity, aneurysmal subarachnoid hemorrhage (aSAH) is the most common cerebrovascular condition and ranks only second to cerebral hemorrhage and cerebral infarction. Overall incidence rate of aSAH is around 6.1 per 100,000 individuals worldwide (1). With a serious prognosis and high mortality rate, aSAH not only provides a great economical burden on the family but burdens society as a whole (2). One of the common complications of aSAH is a delayed cerebral ischaemia attack (DCI), which might result in poor prognosis for aSAH. DCI usually occurs 3–14 days after primary hemorrhage and its prevalence is 29% (3, 4).

It has also been reported that 38.5–65% of patients with aSAH require mechanical ventilation (5–8), while 18–50% develop acute lung injury (9, 10). Accordingly, the treatment of mechanical ventilation is often required in severe aSAH as an additional therapeutic measure. This implies that the lungs and central nervous system are closely related, and that patients with aSAH who need continuous mechanical ventilation often have a worse prognosis (11, 12). In patients with ALI, factors such as impaired systemic vascular endothelial function due to an intense inflammatory response, as well as mechanical ventilation and other therapeutic measures during ALI treatment, may have an impact on brain function, thereby increasing the risk of DCI. The relationship between ALI and DCI is multifaceted and complex, involving several interrelated pathophysiologic processes, which may be at odds with each other in terms of management strategies (13). ALI treatment strategies of high PEEP, permissive hypercapnia due to small tidal volume and prone ventilation may lead to increased intracranial pressure and decreased cerebral blood flow, while measures to increase cerebral perfusion, including volume resuscitation, elevated blood pressure, and hypertonic therapy may interfere with the conservative fluid management strategies recommended for ALI.

At present, the risk factors for DCI in patients with aSAH combined with ALI remain unpredictable, and no risk prediction model has been established. Early identification of risk factors for DCI is particularly important in comatose aSAH patients, and reversal of DCI as soon as possible before the ischemic process progresses to cerebral infarction requires early detection by the clinician, which is a critical step for effective intervention (14). Therefore, early and accurate prediction of DCI and poor prognosis is essential for timely intervention in patients.

Consequently, our objective is to develop and verify a simple, precise, and broadly applicable column line graph. The column line graph prediction model is often used for clinical illness risk assessment, integrating the identification of risk factor predictors and providing an understandable and visual representation of the data prediction outcomes (12). This study retrospectively examined the clinical data of patients with aSAH complicated with ALI post-admission, investigated the risk factors for DCI in this cohort, and developed and validated a column-line diagram prediction model. This model aids clinicians in identifying critically ill patients at risk of DCI during treatment, thereby enhancing patient management and offering a robust theoretical foundation for early clinical intervention and improved prognosis in aSAH patients.

## Materials and methods

### Objects of study

With the following inclusion criteria, 264 patients with aSAH who needed mechanical ventilation and were hospitalized to Lishui People’s Hospital’s Emergency Intensive Care Unit (EICU) and Intensive Care Unit (ICU) between January 2018 and June 2024 were chosen using a retrospective research approach: (1) patients who were ≥ 18 years old, independent of gender; (2) those with aSAH who had also experienced ALI while in the hospital; and (3) patients with aSAH verified by subarachnoid hemorrhage resulting from a ruptured aneurysm, as confirmed by computed tomography angiography (CTA). The following were the criteria for exclusion: The following conditions may be present in patients: (1) drug-induced aneurysm rupture; (2) stroke, brain tumor, or severe head trauma history; (3) severe systemic diseases, including hepatic, renal, cardiac, and hematological disorders; (4) other cerebrovascular malformations, including co-morbidities, such as smoky disease. (5) Individuals who were admitted with insufficient clinical data. Ethical: The hospital’s Ethics Committee accepted this research, which conformed with medical ethical requirements (approval number: 2024–130).

**Figure 1:**
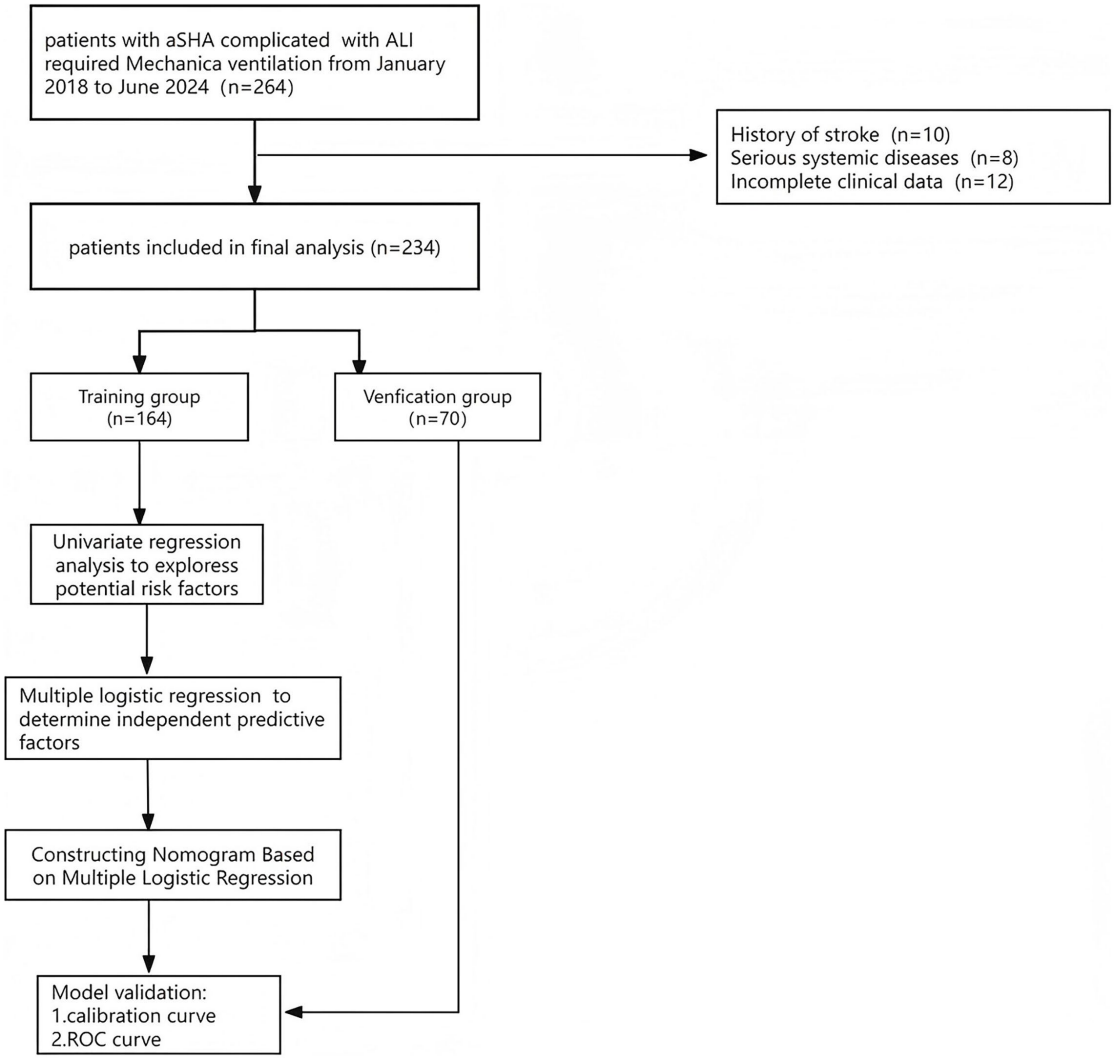
Schematic of patient’s inclusion process and flow chart with the study. Include inclusion and exclusion criteria.

Following the exclusion of 10 patients with a history of stroke or other illnesses, 8 patients with serious systemic disorders, and 12 patients with insufficient admission information, 234 patients were included in the statistical analysis and they were randomly divided into training and validation sets in the ratio of 70%:30% (Figure 1).

### Clinical data collection

The following informations were recorded: (1) Basic information: age, gender, history of alcohol consumption, history of smoking, underlying diseases (e.g., diabetes, cardiovascular disease), etc. (2) Laboratory indicators: Collection of Laboratory indicators within 24 h after aneurysm rupture:white blood cell (WBC), neutrophil percentage, lymphocyte percentage, platelet (PLT), blood urea nitrogen (BUN), blood creatinine (CR), interleukin-6 (IL-6), C-reactive protein (CRP), etc.; (3) Clinical data: HuntHess grade, modified Fisher grading, PEEP value, aneurysm site, parenchymal brain hemorrhage, extraventricular drainage, etc. All patients were randomised to either the training group (164 patients) or the validation group (70 patients). They were also assessed for DCI risk factors and separated into groups with and without DCI based on whether or not DCI occurred while they were in the hospital.

DCI was defined as a new neurological deficit (e.g., hemiparesis, aphasia, apraxia, paraphasia, hemianopsia, or neglect) or a decrease in GCS score of at least 2 points with imaging (CT/MRI) evidence of ischemia, with symptoms persisting for ≥1 h, which usually occurs 3–14 days after the hemorrhage, and which could not be attributed to any other cause (15). Additionally, the PEEP of the ventilator parameters throughout hospitalization was greater than 10 cmH2O for the high PEEP group in this research, while it was lower than 10 cmH2O for the low PEEP group.

### Statistical analyses

Data processing was conducted using SPSS version 26.0 and R software version 4.3.0. Data that followed a normal distribution were presented as mean ± standard deviation (x ± s), and an independent samples *t*-test was used for group comparisons. Measurements that were not normally distributed were represented as *M (Q1, Q3)*, with intergroup comparisons conducted using the rank-sum test. Count data were presented as the number of instances (*n*, %), with comparisons performed using the *χ2* or Fisher exact test. Univariate analysis was used to identify possible risk factors for the onset of delayed cerebral ischaemia in patients, and variables with *p* < 0.05 in the univariate analysis were included into the multivariate logistic regression model. A predictive column chart using independent risk indicators was subsequently created with the “rms” program in R software. Consistency indices (C-index) were computed, Hosmer-Lemeshow goodness-of-fit tests were conducted, and calibration curves were generated to evaluate the model consistency of the column charts. Receiver operating characteristic (ROC) curves were used, and the area under the curve (AUC) was utilized to evaluate the discriminatory efficacy of the column-line diagrams. Furthermore, we contrasted the AUC of the column-line diagram with the AUC of all independent risk factors. Statistical significance was established at *p* < 0.05 (two-sided test), and regression coefficients were presented with 95% confidence intervals (CIs).

## Results

Clinical features of patients in the training and validation groups: 164 patients from the training group and 70 patients from the validation group made up the 234 patients that were analyzed. Interleukin-6, D-dimer, diabetes mellitus, parenchymal hemorrhage, and excessive PEEP were significantly different in the training group when compared to baseline data (*p* < 0.05) (Tables 1, 2).

**Table 1:** Comparison of clinical characteristics between non-DCI and DCI groups.

Characteristic	Delayed cerebral ischaemia		*p*-value
	No (*N* = 126)	Yes (*N* = 38)	
Male [cases (%)]	59 (46.83%)	17 (44.74%)	0.821
Age (years)	55 (50, 62)	57 (51, 63)	0.407
Drinking history	75 (59.52%)	24 (63.16%)	0.688
Diabetes	38 (30.16%)	28 (73.68%)	<0.001
Cardiovascular disease	65 (51.59%)	13 (34.21%)	0.06
Aneurysm location anterior circulation	106 (84.13%)	34 (89.47%)	0.414
Parenchymal haematoma	50 (39.68%)	22 (57.89%)	0.047
Ventricular drain	84 (66.67%)	28 (73.68%)	0.415
Improved fisher grading			0.756
Class I	1 (0.79%)	0 (0.00%)	
Class II	1 (0.79%)	1 (2.63%)	
Class III	74 (58.73%)	23 (60.53%)	
Level IV	50 (39.68%)	14 (36.84%)	
HuntHess classification			0.162
Class I	1 (0.79%)	0 (0.00%)	
Class II	8 (6.35%)	2 (5.26%)	
Class III	73 (57.94%)	15 (39.47%)	
Level IV	38 (30.16%)	16 (42.11%)	
Level V	6 (4.76%)	5 (13.16%)	
High PEEP	23 (18.25%)	29 (76.32%)	<0.001
Poor prognosis	80 (63.49%)	28 (73.68%)	0.246

PEEP, positive end-expiratory pressure.

**Table 2:** Comparison of laboratory indicators between non-DCI and DCI groups.

Characteristic	Delayed cerebral ischaemia		*p*-value
	No (*N* = 126)	Yes (*N* = 38)	
WBC (10^9/L)	12 (8, 17)	13 (8, 19)	0.625
Percentage of neutrophils	0.82 (0.72, 0.90)	0.82 (0.75, 0.90)	0.864
Percentage of lymphocytes	0.10 (0.06, 0.19)	0.13 (0.07, 0.20)	0.681
Neutrophil-to-lymphocyte ratio	8 (4, 14)	7 (4, 13)	0.749
PLT (10^9/L)	231 (193, 281)	242 (168, 287)	0.592
Cr (umol/L)	60 (51, 77)	58 (51, 88)	0.98
Urea nitrogen (mmol/L)	3.95 (3.03, 5.00)	3.60 (2.90, 4.78)	0.347
Fibrinogen (g/L)	2.69 (2.32, 3.17)	2.73 (2.27, 3.19)	0.997
IL-6 (ng/ml)	7 (2, 12)	14 (6, 19)	0.001
D-dimer (ug/L)	567 (381, 1,097)	2,424 (557, 6,797)	<0.001
CRP (mg/L)	69 (56, 123)	80 (62, 137)	0.48
Albumin (g/L)	33.0 (32.0, 36.0)	34.0 (31.0, 36.0)	0.948
Blood sodium (mmol/L)	141 (134, 148)	137 (135, 141)	0.148
Neuron-specific enolase (ng/ml)	19 (13, 42)	23 (16, 45)	0.359

WBC, white blood cell; PLT, platelet; BUN, blood urea nitrogen; Cr, creatinine; IL-6, interleukin-6; CRP, C-reactive protein.

### Analysis of independent risk factors for the development of DCI after aSAH combined with ALI

High PEEP, interleukin-6, and D-dimer were identified as independent risk factors (*p* < 0.05) using one-way logistic regression employing variables with significant differences in baseline comparison findings (*p* < 0.05). These variables were then integrated in a multifactorial logistic regression model (Table 3).

**Table 3:** Multivariable logistic analysis.

	OR	95% CI	*p*
Diabetes			0.215
Parenchymal haematoma			0.32
High PEEP	16.37	2.36–113.57	0.005
Interleukin-6	1.1	1.02–1.19	0.013
D-dimer	1.74	1.12–2.69	0.014

PEEP, positive end-expiratory pressure; IL-6, interleukin-6.

### Predictive model construction for the occurrence of DCI columnogram after aSAH combined with ALI

**Figure 2:**
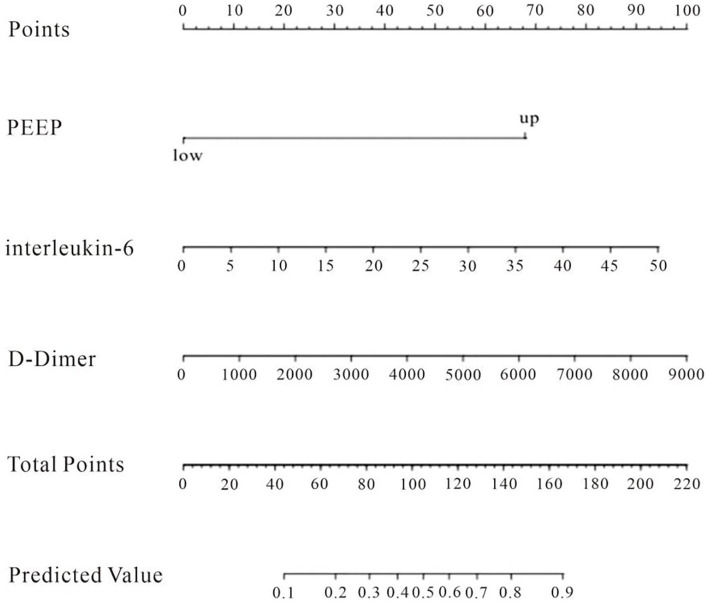
Nomogram for predicting DCI constructed on the basis of 3 independent risk factors.

The factors that met the significance criteria were used to create a line graph prediction model for the probability of DCI after aSAH in conjunction with ALI, as seen in Figure 2. The line graph illustrates that each predictor variable is vertically aligned with a specific value on the “points” axis, while the cumulative scores of all predictors yield a total score that corresponds to the “risk” axis, indicating the patient’s risk of DCI; thus, a higher total score signifies an increased risk of DCI for the patient. The total score reflects the value on the “Predicted Value” axis, indicating the patient’s risk of DCI; a greater total score signifies an increased risk of DCI, while a lower score indicates a reduced risk (Figure 2).

### Performance of column line charts

**Figure 3:**
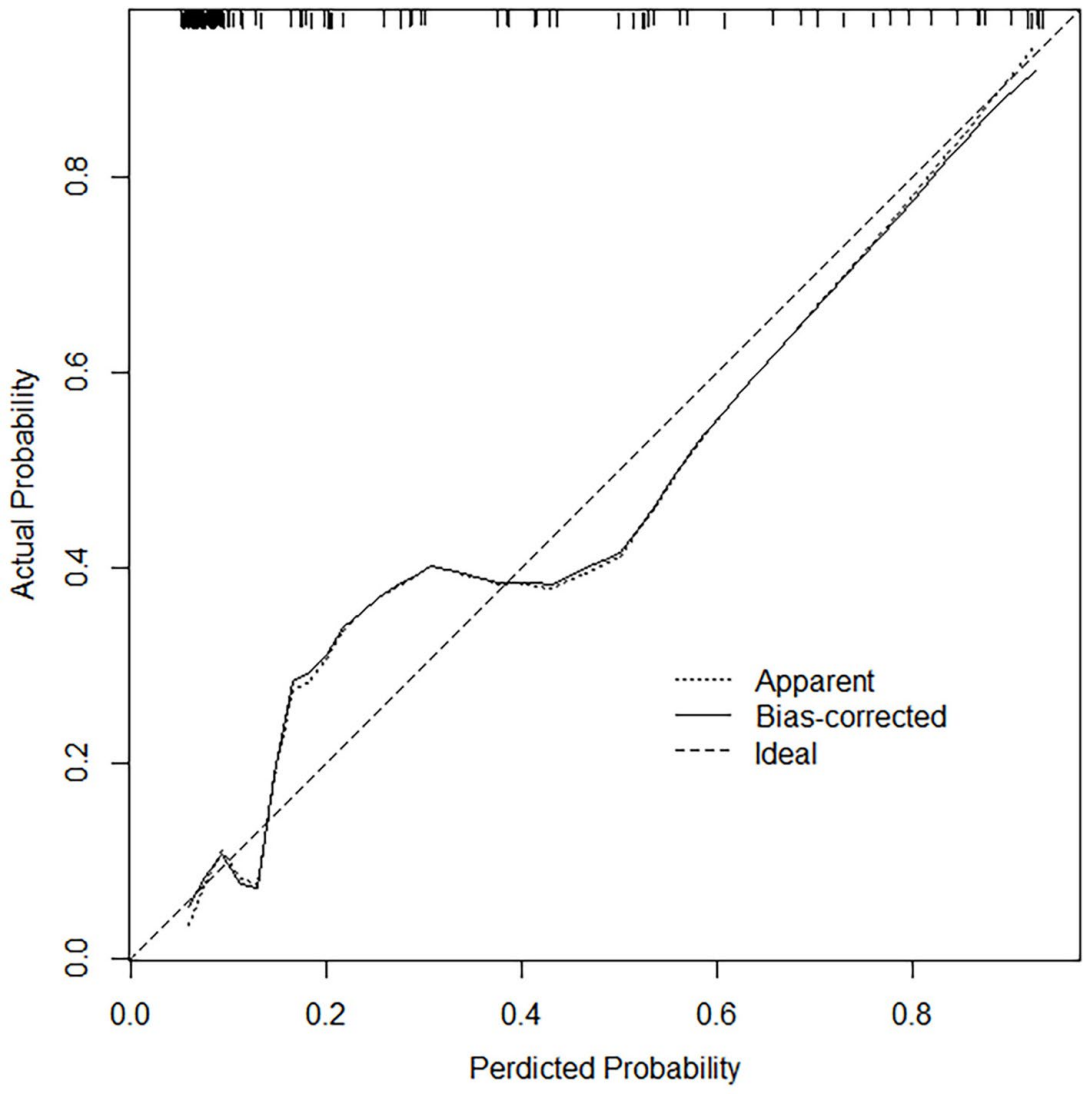
Calibration curves in the training set, the calibration curves mostly fluctuated about the diagonal line, indicating that the model fit was adequate.

**Figure 4:**
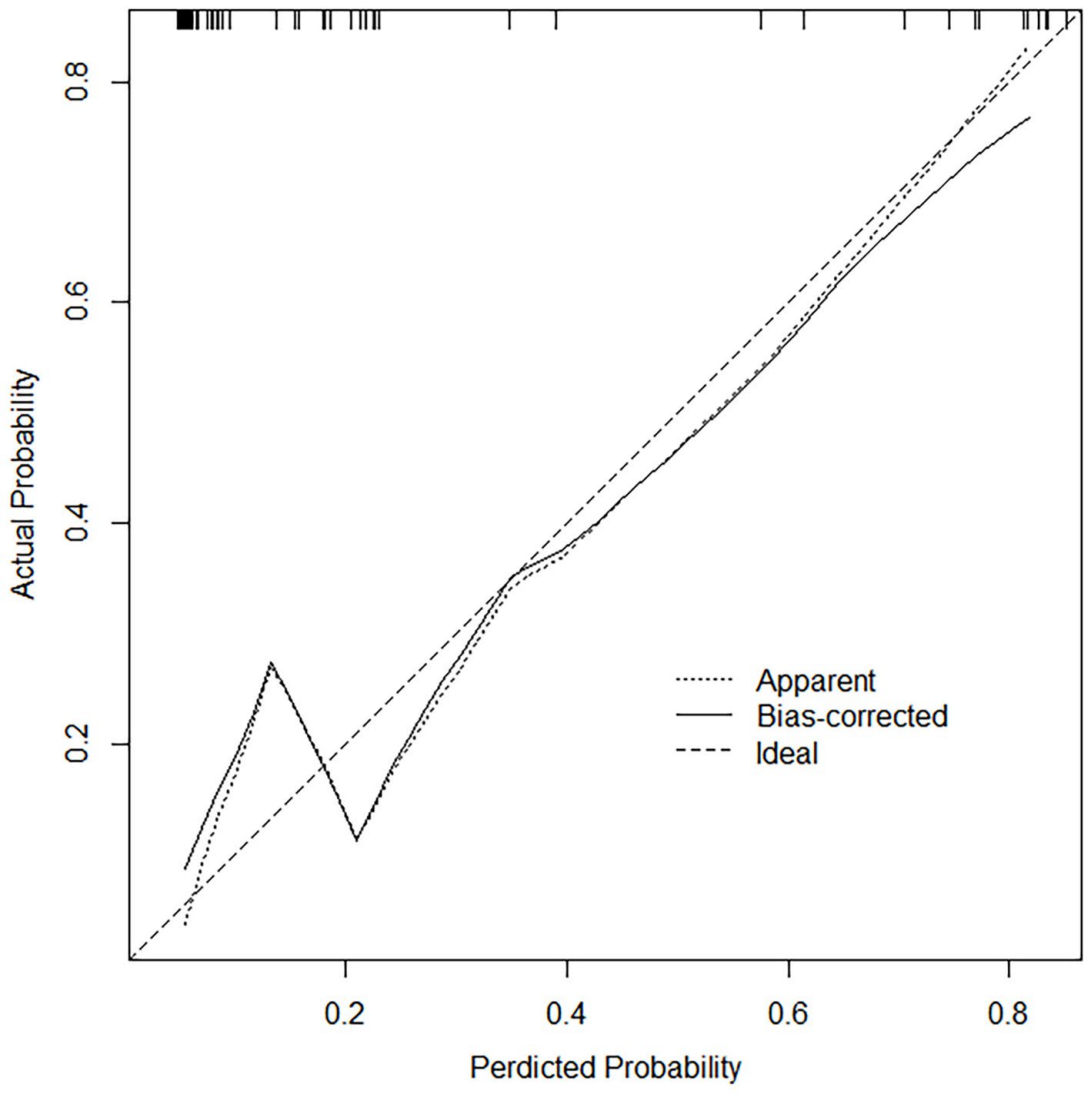
Calibration curves in the validation set, the calibration curve does not deviate from the diagonal, indicating that the model predicted probability is highly consistent with the actual occurrence rate.

For both data sets, the column-line plots’ calibration curves demonstrate a high degree of agreement between the observations and forecasts (Figures 3, 4). The calibration curves mostly fluctuated about the diagonal line, indicating that the model fit was more adequate, while the HL test results for the training set indicated X-squared = 10.539, *p*-value = 0.2292. A stronger validation of the model is shown by the calibration curve result of the validation set, which shows X-squared = 6.951, df = 8, *p*-value = 0.5419, and a curve that essentially changes around the diagonal line (Figures 3, 4).

**Figure 5:**
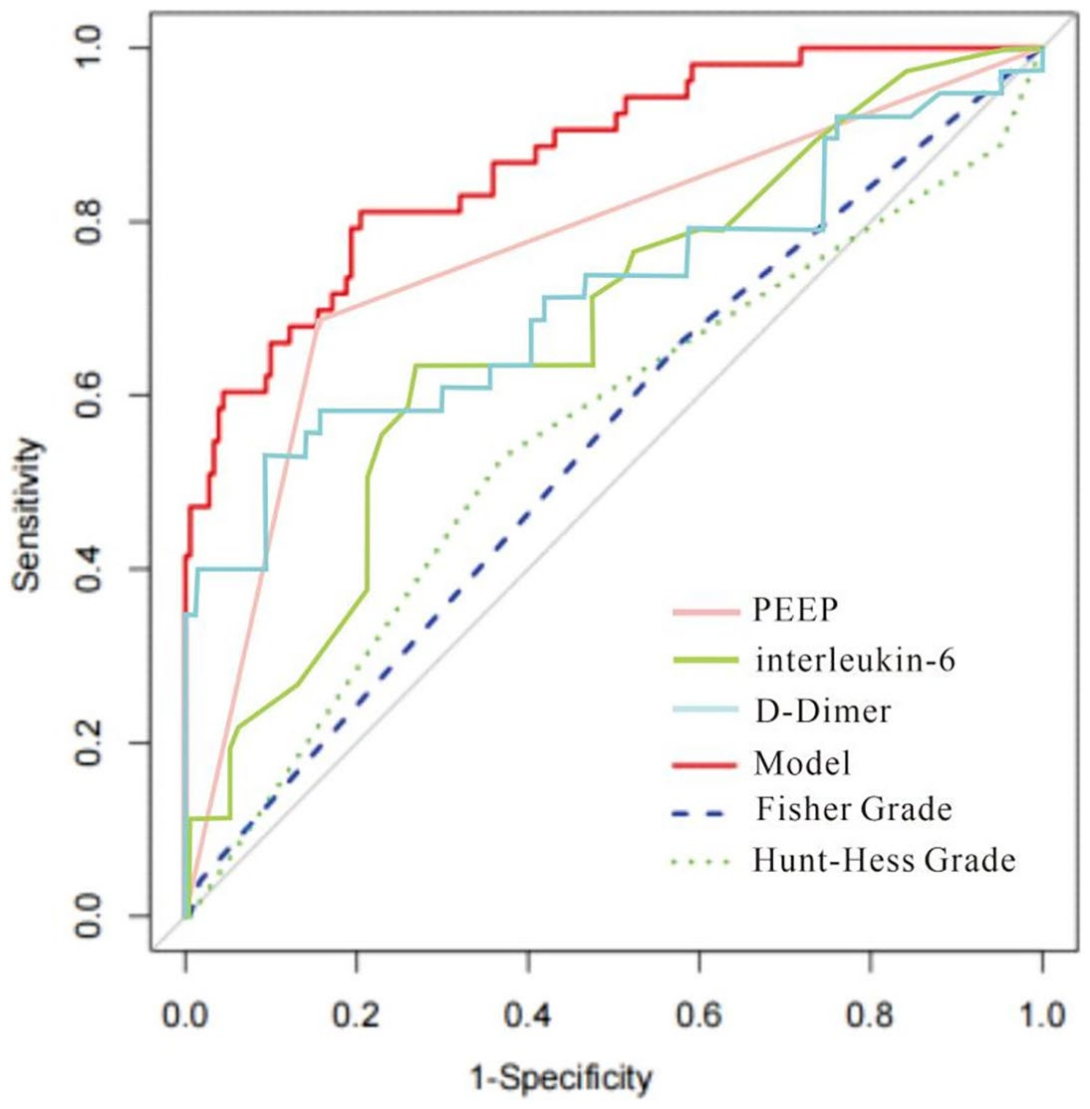
The receiver operating characteristic (ROC) curve of the nomogram in the training set. The AUC for the training set was 0.882 (95% CI: 0.820–0.940); Comparisons with other risk variables in the training set and with traditional predictive models are more favorable.

**Figure 6:**
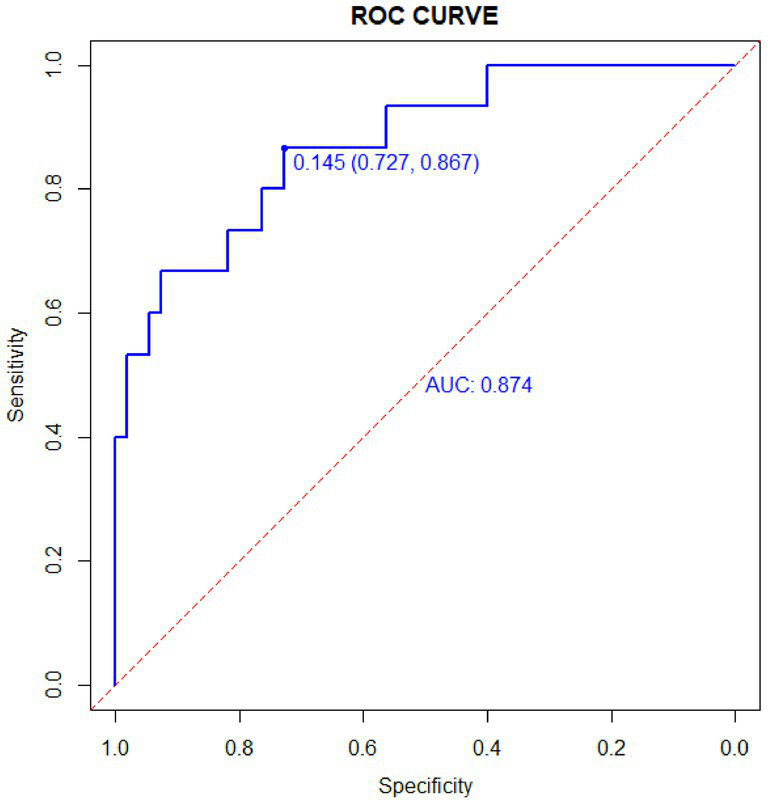
ROC in the validation set. The AUC for the validation set was 0.874 (95% CI: 0.788–0.996).

The column-line diagram’s AUC in the training set was 0.882 (95% CI: 0.820–0.940) (Figure 5), while internal validation in the validation set (Figure 6) yielded an AUC of 0.874 (95% CI: 0.788–0.996), indicating a higher level of discriminating performance. Furthermore, compared to other risk variables in the training set, such as high PEEP (AUC: 0.790, 95% CI: 0.710–0.870), interleukin-6 (AUC: 0.670, 95% CI: 0.570–0.770), and D-dimer (AUC: 0.710, 95% CI: 0.600–0.820), the column-line diagram’s AUC discriminatory power was greater. It is also superior to traditional predictive models (modified Fisher grading, Hunt-Hess grading) (Figure 5).

## Discussion

In this study, we investigated the relationship between early laboratory markers (within 24 h of aneurysm rupture), clinical information, etc., and DCI in 234 patients with aSAH combined with ALI. We then developed and internally validated a predictive column chart for DCI. This column chart modeled that the higher the patient’s interleukin-6 and D-dimer values and the higher the level of PEEP, the higher the patient’s risk of DCI. This column chart is superior to traditional prediction systems, such as Fisher Grade and Hunt-Hness Grade, in predicting the occurrence of DCI, and has good clinical value.

In clinical practice, combined acute lung injury in aSAH is not unusual; according to one study, ALI was diagnosed in 50% of aSAH patients and 45% of patients on the first day of mechanical ventilation (MV) therapy (16), and the development of ARDS was independently correlated with the severity of aSAH disease and clinical hemorrhage grading (10). The mechanism causing lung injury in patients with acute SAH is a “double whammy” model, wherein the first whammy is caused by the adrenergic surge and systemic inflammation brought on by acute neurological injury, and the second whammy is caused by non-neurological stressors like infections, transfusions, and MV (17–19). One important pathophysiological characteristic of ALI is alveolar collapse, which may result in hypoxaemia brought on by intrapulmonary shunts. One approach to preserve functional residual capacity and enhance oxygenation in ALI is alveolar reopening, which involves opening collapsed lung units using PEEP. One of the main causes of aneurysmal subarachnoid hemorrhage’s bad prognosis is delayed cerebral ischaemia. Once DCI occurs, its effects are severe, permanent, and linked to a short-term death rate, poor result, and a severe clinical course (1). However, to date, very little can be done to prevent or treat DCI after aSAH (20). On the one hand, this is related to the complex mechanisms of DCI, which has been considered to be mainly caused by cerebral vasospasm, and then recent studies have emphasized the role of microthrombi, coagulation and fibrinolytic systems, neuroinflammation, and cortical speading depolarization in DCI (21). On the other hand, it may also be attributed to the clinical failure to accurately predict and recognize DCI. In the present study population, mechanically ventilated patients often cannot be assessed by a reliable clinical neurological examination in the same way as lucid patients, and therefore it is necessary to search for reliable biological markers or clinical tools to predict the occurrence of DCI.

High PEEP was one of the independent risk variables in this research for developing DCI in those patients who had aSAH complicated with ALI. Since cerebral vasospasm most often occurs between days 6 and 8, earlier studies have identified day 7 after bleeding as an important juncture in the typical course of aSAH (22). On day seven post hemorrhage, ICP was significantly higher and the MAP from the baseline lower in the group with a PEEP of 20 cmH2O compared to the group with 5 cmH2O. This was later followed by a decline in cerebral blood flow, which leads to an increased risk of DCI (22, 23). However, there are conflicting findings regarding the effect of PEEP on intracranial pressure. In some studies in SAH, no significant effect on cerebral blood flow (CPP) was observed when PEEP was increased to 15 cmH2O (23, 24). A significant relationship between PEEP and ICP, CPP was found only when severe brain injury occurred. Each 1 cm H2O increase in PEEP resulted in a 0.31 mmHg increase in ICP and a 0.85 mmHg decrease in CPP (25). The European Society of Intensive Care Medicine (ESICM) guidelines for the management of ventilation in patients with acute brain injury conclude that the use of lung protection strategies in patients with combined acute respiratory distress syndrome (ARDS) and acute brain injury without significant intracranial pressure (ICP) elevation is “strongly recommended but not proven.” However, there is no consensus on whether lung-protective ventilation should be used in patients with ARDS who have combined brain injury and clinically significant elevated intracranial pressure (26).

In the past 2 years, the generalization of IL-6 testing has become more and more widespread around the world, especially in some developed regions such as the United States, Europe, and Japan, etc. In our country, many large hospitals and research institutes have incorporated IL-6 testing into their routine screening programs, especially in the fields of intensive care, infectious diseases, and autoimmune diseases. High IL-6 levels have also been associated with the occurrence of DCI (27, 28). Throughout the whole aSAH damage mechanism, inflammation takes place. When an aneurysm bursts, a lot of inflammatory cells enter the subarachnoid space, quickly triggering an inflammatory response, and the blood cells that are deposited there stimulate the brain tissue and activate the central nervous system’s immunoregulatory cells. On the other hand, ALI causes brain injury through mechanisms such as inflammation, hypoxemia, and adverse effects of mechanical ventilation, and ALI causes neuroinflammation by inducing excessive release of pro-inflammatory cytokines, which promotes the onset and development of cerebral vasospasm. A number of studies have investigated the potential correlation between elevated IL-6 levels and DCI and found that IL-6 levels usually peak in the first week after hemorrhage, which is the most likely time point for DCI, suggesting that increased IL-6 may be associated with delayed onset of symptomatic vasospasm (29).

According to the study’s findings, patients in the DCI group had greater D-dimer values than those in the non-DCI group. An elevated level of D-dimer, a key component of the body’s coagulation system and the breakdown product of fibrin activation and hydrolysis, may indicate the presence of secondary hyperfibrinolytic functions in the body, such as disseminated intravascular coagulation, which can impact the blood supply of cerebral blood vessels and result in DCI. Many studies have shown changes in both coagulation and fibrinolytic cascade responses after SAH (30, 31). D-dimer primarily reflects the function of fibrin solubilization. Ischaemic cerebrovascular disease (ICVD) is strongly associated with significantly increased D-dimer (32, 33). According to some research (34), Serum D-dimer levels over 445 μg/L have been proposed as a predictor of delayed cerebral ischaemia in Fisher class IV patients with aneurysmal subarachnoid hemorrhage.

In summary, elevated PEEP, interleukin-6, and D-dimer are independent risk factors for DCI in aSAH patients complicated with acute lung damage. The aforementioned three indications may be acquired by the collection of medical history and standard laboratory tests, which are feasible and straightforward. The model demonstrated strong predictive power, this will help clinicians better identify at-risk populations and help them make appropriate clinical decisions. For example, if a patient is determined to be at high risk of developing a DCI based on a line drawing, early measures must be taken. Examples include transcerebral perfusion imaging to determine if the patient is experiencing cerebral vasospasm, as well as the use of nimodipine to prevent and maintain normal cerebral vascular volume, more aggressive management of systemic inflammation, coagulation system or early and effective neuroprotective measures. Careful titration of PEEP during mechanical ventilation based on pulmonary compliance, and strict maintenance of MAP to balance the dual demands of oxygenation and cerebral blood flow. Non-invasive methods such as transcranial Doppler (TCD), pupillometry and optic nerve sheath diameter (ONSD), lung and brain ultrasound should be used to guide PEEP titration. And in low-risk patients, overdosing can be avoided.

To date, the prevention and treatment of DCIs remains a critical and complex issue in the treatment of aSAH, and nimodipine is the only drug that can reduce the incidence of DCIs. In the case of the predictive model in this study, while it facilitates the early identification of high-risk patients, direct demonstration of its effectiveness in reducing mortality does present some challenges and the interventions available are relatively limited. The parameters chosen for this study reflect the level of inflammation and coagulation status of the patients, but did not include indicators of endothelial dysfunction (one of the causative mechanisms of DCI). The results of the study reflect risk factors for DCI in only a subset of the population, but it seems difficult to generalize to all aSHA. In addition, possible biases in the interpretation of D-dimer in patients with venous thromboembolism were not excluded in this study, which would have had some impact on the accuracy of the model. Sensitivity analyses could be performed in the future to further understand the robustness of the findings.

## Conclusion

The current research concluded that the incidence of DCI in aSAH patients complicated with ALI was linked to raised PEEP, as well as increased levels of interleukin-6 and D-dimer. The column-line graph developed in this research effectively predicted the incidence of DCI in patients with aSAH and ALI. Based on these results, focused programs might be offered in clinical practice to improve comprehensive monitoring and treatment to avoid DCI.

## Data Availability

The original contributions presented in the study are included in the article/supplementary material, further inquiries can be directed to the corresponding author.
